# Benchmarking Large Language Models for MIMIC-IV Clinical Note Summarization

**DOI:** 10.1007/s41666-025-00221-9

**Published:** 2025-11-05

**Authors:** Amin Naemi, Ali Sahafi

**Affiliations:** 1 Department of Biology, University of Southern Denmark, Campusvej 55, Odense, 5230 Denmark; 2 Institute of Mechanical and Electrical Engineering, University of Southern Denmark, Campusvej 55, Odense, 5230 Denmark

**Keywords:** Large language model, Text summarization, Artificial intelligence, Deep learning, MIMIC

## Abstract

Large Language Models (LLMs) are increasingly applied in healthcare and are expected to play an active role in clinical practice. However, their effectiveness for clinical note summarization remains underexplored, and systematic comparisons across different models are lacking. This study addresses this gap by benchmarking 16 generative LLMs from major providers, including OpenAI (GPT), DeepSeek, Meta (LLaMA), Google (Gemma), Mistral (Mixtral), and Alibaba (Qwen), using the MIMIC-IV-Note. Both extractive and abstractive summarization approaches were implemented and evaluated with multiple lexical and semantic metrics, including ROUGE, BLEU, METEOR, COMET, and BERTScore. In addition, processing time, cost, and deployment feasibility were assessed to provide a practical perspective for clinical adoption. The results show that Gemma-3-27B achieved the strongest overall performance in extractive summarization. For abstractive summarization, DeepSeek-R1-70B, Qwen-3-32B, and GPT-4o emerged as leading models. Their relative strengths varied depending on whether lexical overlap, semantic adequacy, or fluency was prioritized. Importantly, larger parameter sizes did not always translate into better outcomes, as smaller models such as LLaMa-3-8B and Gemma-2-9B often produced competitive results with faster runtimes and lower computational costs. This study highlights the trade-offs between performance, efficiency, and deployment context that offers practical insights into model selection for clinical note summarization and informing future integration of LLMs into healthcare workflows.

## Introduction

In the healthcare domain, vast amounts of patient records, medical notes, and clinical documentation are generated daily, providing a wealth of information for medical decision-making and research. However, manually extracting relevant insights from these records is a time-consuming and labor-intensive task. A study has shown that primary care physicians spend nearly half of their 11.4 hours workday on Electronic Health Records (EHR) related tasks [1]. Clinical notes, as an unstructured component of EHR, are rich in detail but often lengthy and difficult for healthcare professionals to quickly extract key information from. Therefore, automatic text summarization is a prominent area of research in information retrieval, especially within the medical and biomedical fields. It provides an effective solution to manage the increasing volume of scientific and clinical literature by condensing source documents while preserving their key information. In other words, text summarization helps distill essential information from patient records while preserving the integrity and precision of the information [2, 3].

With the advent of Artificial Intelligence (AI), automated text summarization has seen remarkable advancements. AI-driven approaches, particularly those based on Natural Language Processing (NLP) and Deep Learning (DL), have enabled the efficient extraction of relevant medical information while reducing the cognitive burden on healthcare professionals [4].

Large Language Models (LLMs) have played a pivotal role in this transformation, as they demonstrate remarkable capabilities in text generation, summarization, and comprehension across diverse domains [4]. In other words, LLMs have revolutionized text processing by leveraging transformer-based architectures to generate human-like text with remarkable accuracy and contextual awareness. These models undergo pre-training on extensive datasets followed by fine-tuning for domain-specific tasks, which makes them highly effective across various fields. The self-attention mechanisms in transformer architectures allow LLMs to capture complex linguistic patterns, maintain contextual dependencies, and generate coherent summaries, which make them invaluable for processing large-scale unstructured medical data. Their ability to identify key information, distill relevant insights, and generate concise summaries has positioned them as a transformative tool for improving efficiency in healthcare applications.

In recent years, several AI research labs and technology companies have played a pivotal role in developing LLMs. GPT is a family of AI language models developed by OpenAI. It is based on the transformer architecture, originally introduced by Vaswani et al. in 2017 [5]. Similarly, Meta has also introduced LLM models called LLaMA. These models are designed for efficiency and accessibility, so these models are adopted for research and enterprise applications [6]. Google DeepMind’s Gemma models focus on lightweight, high-performance architectures optimized for structured response generation and instruction-following tasks [7]. DeepSeek’s transformer-based models emphasize multilingual capabilities and contextual depth. DeepSeek employs a carefully optimized training strategy that incorporates reinforcement learning from human feedback and fine-tuning methodologies to enhance its contextual awareness and response accuracy. Compared to other models, DeepSeek is engineered for broader generalization capabilities while maintaining strong factual consistency and reliability [8]. Its architecture supports improved long-context comprehension, which makes it highly effective for applications in research, healthcare, and technical documentation [9, 10]. Meanwhile, Mistral AI’s Mixtral models introduce the Mixture of Experts (MoE) mechanism, where only a subset of parameters is activated per input. This approach significantly enhances computational efficiency without compromising performance [11]. Alibaba has also introduced its LLM model called Qwen which utilizes advanced DL techniques to focus on scalability, efficiency, and semantic understanding for a variety of NLP tasks [12].

These LLMs are large-scale DL models trained on an extensive corpus of text data using unsupervised learning techniques. These models network predict the likelihood of a word or phrase appearing in a given context, which enables it to generate coherent and contextually relevant text [13].

These advancements demonstrate how LLMs are reshaping the landscape of AI-driven text processing. In the medical domain, LLMs have the potential to assist healthcare professionals with tasks such as patient diagnosis [13], treatment decision-making [14], improving radiology report analysis [15], reviewing clinical reports [16], educating patients [17], and text summarization [18]. However, the process of generating accurate, concise summaries remains a challenge, particularly when it comes to balancing precision and fluency.

Therefore, given the importance of automated summarization tools in clinical practice, this study aims to assess the performance of well-known generative LLMs for both extractive and abstractive summarization of clinical notes from the MIMIC-IV-Note database. We evaluate the models based on performance metrics as well as time and cost factors to offer a comprehensive comparison. This work not only advances the field of clinical NLP but also provides valuable insights into model efficiency, scalability, and practical applicability for healthcare professionals.

## Materials and Method

**Fig. 1:**
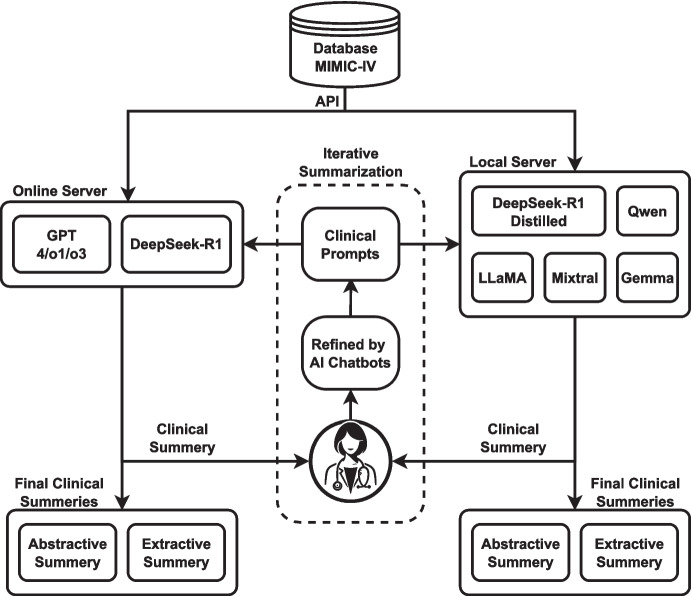
Workflow diagram of this study

This section provides an overview of the dataset and the methodological framework of the study. LLMs vary in their accessibility and deployment methods, including online API-based access, locally hosted models, and other implementation strategies. Figure 1 illustrates the study’s methodology, outlining the process of integrating and utilizing different LLMs for text summarization.

In our study, we explored two approaches for utilizing LLM models, including accessing online servers or running them on a local server (Fig. 1). In an iterative process, we asked clinicians to review both the summaries and the clinical prompts fed to the LLM models, while also refining the prompts using AI chatbots. Through this iterative approach, we identified the optimal clinical prompts for feeding into the LLM models. Finally, we instructed the LLMs to generate both abstractive and extractive summaries for all patients.

### Dataset and Preprocessing

In this study, we used MIMIC-IV-Note data which includes a comprehensive collection of over 2.6 million deidentified clinical notes recorded during patient admissions to Intensive Care Units (ICUs) at Beth Israel Deaconess Medical Center. These unstructured free-text documents capture essential patient information, including 331,794 discharge summaries from 145,915 patients and 2,321,355 radiology reports for 237,427 patients. The dataset also contains progress notes, nursing documentation, electrocardiogram interpretations, and physician reports [19].

To ensure patient privacy, all notes have been anonymized in accordance with the Health Insurance Portability and Accountability Act (HIPAA). The dataset is structured to improve data accessibility and interoperability, which enhances its utility for both research and real-world applications [20].

MIMIC-IV-Note provide a comprehensive record of a patient’s hospitalization, from admission to discharge. These notes include basic demographic information, such as name, admission dates, and service, as well as critical details like allergies, chief complaints, and the history of present illness. They also capture the patient’s past medical history, physical examination findings, and relevant lab results, which inform diagnosis and treatment plans.

Throughout the hospitalization, the notes document procedures, medications, and the patient’s progress. This includes any surgeries or interventions performed, alongside ongoing assessments of the patient’s condition. The discharge summary outlines the patient’s status at the time of release, including follow-up care instructions and prescribed medications.

In this study, we randomly selected 30,000 samples from the MIMIC-IV-Note clinical notes dataset and tasked the LLM models with generating summaries for each sample.

### LLM Models

In this study, we apply a range of well-known cloud-based and local LLMs. To ensure consistency across all models, the temperature parameter is set to 0. This configuration removes randomness from the generation process and guarantees reproducible outputs.

#### GPT

GPT models, developed by OpenAI, are transformer-based LLMs that are used in multimodal reasoning and long-context processing [21, 22].

GPT-4-Turbo, launched in November 2023, is an optimized and cost-effective variant of GPT-4 with a 128K context window. It delivers faster responses and reduced resource consumption while preserving high-quality outputs [23, 24]. GPT-4o, released in May 2024, enhances reasoning ability, contextual understanding, and multimodal performance by processing both text and images [22]. GPT-o1-mini, introduced in September 2024, is a lighter version of GPT-o1, which was designed for scientific reasoning, mathematics, and coding. It uses advanced optimization to reduce computational costs and latency while maintaining strong performance for real-time and resource-constrained applications [25]. GPT-o3-mini, released in January 2025, further improves efficiency with a compact design that balances speed and accuracy. This model is suitable for smaller-scale clinical tasks where rapid responses and low overhead are essential [26].

#### DeepSeek

DeepSeek is a family of transformer-based LLMs designed for complex linguistic tasks with strong contextual understanding [27].

DeepSeek-R1-671B is an open-weight model trained on large multilingual datasets, excelling in scientific, technical, and conversational applications. DeepSeek-R1-70B is a distilled variant that integrates Meta’s LLaMA-70B architecture to reduce computational cost while retaining strong reasoning ability. It is designed for efficiency and real-time use. It performs well in summarization and conversational tasks while being suitable for deployment in resource-limited environments such as edge devices and clinical systems with strict latency requirements [28].

#### LLaMA

LLaMA, developed by Meta AI, is a family of open-weight transformer-based models designed to balance performance, scalability, and accessibility [6].

LLaMa-3.3-70B is a large-scale model with 70 billion parameters, designed for versatility across diverse domains. Its architecture supports complex input processing and high-quality outputs without extensive fine-tuning [29]. LLaMa-3-8B is a compact model with 8 billion parameters that offers strong efficiency for processing longer sequences, making it suitable for real-time applications and resource-constrained environments [30].

#### Gemma

Gemma is a family of lightweight, high-performance open-weight transformer-based models developed by Google DeepMind [7].

Gemma-3-27B, released in March 2025, introduces multimodal capabilities that support over 140 languages. By utilizing distillation and reinforcement learning, it offers strong reasoning, multilingual performance, and efficient deployment on a single GPU or TPU [31]. Gemma-3-21B offers a balance between capability and efficiency that means it requires less computational resources than Gemma-3-27B [31]. Gemma-2-9B is a 9B parameter instruction-tuned variant from the Gemma-2 series that is optimized for structured response generation and guided reasoning [32].

#### Mixtral

Mixtral is a family of MoE language models developed by Mistral AI. They achieve high performance with improved computational efficiency by activating only a subset of expert pathways per token [11].

Mixtral-8x22B is a larger MoE variant that uses eight 22B parameter experts. This design delivers significant efficiency and elevated performance across benchmarks [33]. Mixtral-8x7B is a sparse MoE model with eight 7B parameter experts and two active per token. It achieves high accuracy while benefiting from reduced computational cost and scalability [11].

#### Qwen

Qwen is a family of large language models developed by Alibaba Cloud. They are known for strong multilingual abilities, long-context processing, and specialized variants such as coding-focused models [34].

Qwen-2.5-32B supports up to 131,072 tokens and achieved a strong 65.9 on the McEval programming benchmark [12, 35]. Qwen-3-32B is a dense 32B model that advances hybrid reasoning and agentic capabilities that offer broad multilingual support across 119 languages and deliver efficient performance in complex tasks like code generation [36]. Qwen-3-8B is a compact dense model that incorporates grouped query attention and architectural optimizations to support efficient long-context handling and hybrid reasoning tasks [36].

### Server Specifications

In this study, the GPT models and the DeepSeek-R1-671B model were accessed exclusively via online APIs. In contrast, the other models, including DeepSeek-R1-70B, LLaMa-3.3-70B, LLaMa-3-8B, Gemma-3-27B, Gemma-3-12B, Gemma-2-9B, Mixtral-8x22B, Mixtral-8x7B, Qwen-2.5-32B, Qwen-3-32B, and Qwen-3-8B ran locally on a server configured with Ollama-0.5.11, dual AMD EPYC 7F72 24-core processors (48 threads at 3.19 GHz), and an NVIDIA A100 PCIe GPU (40 GB).

### Extractive and Abstractive Summarization

Extractive and abstractive summarization are two primary approaches for generating summaries from large bodies of text. Extractive summarization selects and directly extracts key sentences or segments from the original text to form a concise summary, that ensures the extracted content is verbatim from the source. In contrast, abstractive summarization involves generating new sentences that paraphrase and rephrase the original content. It often uses advanced NLP techniques to produce more human-like, coherent summaries. While extractive methods rely on sentence selection, abstractive methods aim to capture the essence of the text while producing novel sentence structures. Both techniques have been widely used in various applications, including document summarization and clinical note analysis [37]. We refined our methodology through an iterative process. The final prompts are presented below.

- **Extractive Prompt:**

*You are a Clinical Assistant with two tasks.*

*Task 1: Create a section titled “Summary of Patient” and summarize the following patient journal by extracting and retaining all key clinical details in a structured, cohesive narrative. The summary should be written as a single paragraph of at least 300 words, avoiding bullet points. Ensure that it closely mirrors the original text’s language while maintaining clarity and readability.*

*Task 2: First extract all headings from the patient journal. Then for each heading, if the information is provided as bullet points (e.g., Medications), copy it verbatim. If the information is in sentence format, generate a concise summary while preserving the original text’s tone and phrasing.*

- **Abstractive Prompt:**

*You are a Clinical Assistant, and a patient’s clinical data is provided for you.*

Include a section with the name of *Summary* and summarize the clinical notes in a 200-word paragraph using only the provided details.

*Then, add separate bullet-point sections for:*

*Diseases*: List all patient illnesses.

*Family Background*: List all family background details.

Include a section for *Admission Medications*.

Include a section for *Discharge Medications*.

Include a section for *Discharge Instructions*.

*Do not include any details not explicitly stated.*

### Evaluation Metrics

Evaluating text summarization models ensures they generate concise, relevant, and accurate representations of the original content. Traditional metrics focus on lexical overlap, measuring precision, recall, and similarity between generated and reference summaries. However, abstractive methods often require semantic similarity metrics to assess meaning beyond exact word matches. Therefore, in this study, we consider a variety of performance metrics to evaluate the performance of models in providing summaries.

#### ROUGE

Recall-Oriented Understudy for Gisting Evaluation (ROUGE) is a key metric for evaluating text summarization by measuring the similarity between generated and reference summaries. It primarily assesses recall, capturing how much of the reference text appears in the summary.

ROUGE-N evaluates n-gram overlap, with ROUGE-1 measuring unigram matches and ROUGE-2 capturing bigram similarit. ROUGE-L goes beyond word overlap by using the longest common subsequence to assess fluency and coherence, which benefits abstractive summarization by evaluating the preservation of meaningful word sequences in the summary. These metrics are essential in understanding how well the generated summary aligns with the reference summary in terms of both lexical overlap and structural coherence [38].

#### BLEU

Bilingual Evaluation Understudy (BLEU) is a precision-based metric that evaluates text generation by measuring n-gram overlap between the generated and reference summaries. It calculates precision scores for unigrams, bigrams, and higher-order n-grams while applying a brevity penalty to prevent overly short outputs from inflating scores [39]. Unlike ROUGE, which emphasizes recall, BLEU focuses on how much of the generated text matches the reference. The BLEU score is computed as:

$$\text {BLEU} = BP \times \exp \left( \sum _{n=1}^{N} w_n \log P_n \right)$$

where $P_n$ represents *n-gram precision*, $w_n$ is the *weight assigned to each n-gram order*, and $BP$ is the *brevity penalty* to discourage overly short summaries.

#### METEOR

Metric for Evaluation of Translation with Explicit ORdering (METEOR) improves upon BLEU by considering synonyms, stemming, and word order in text evaluation. Unlike BLEU, which relies solely on n-gram precision, METEOR aligns words semantically by allowing variations such as inflected forms and synonyms. It calculates precision, recall, and alignment scores while applying a fragmentation penalty to penalize disordered word sequences [40]. The METEOR score is computed as follows:

$$\text {METEOR} = F_{\text {mean}} \times (1 - \text {Penalty})$$

where $F_{\text {mean}}$ represents the *harmonic mean of precision and recall*, balancing both measures to ensure an accurate evaluation of summary quality. The *Penalty* term accounts for *word order mistakes*, reducing scores for summaries that fail to maintain proper structure and coherence.

#### COMET

Crosslingual Optimized Metric for Evaluation of Translation (COMET) is a neural-based evaluation metric designed to assess the quality of text generation tasks, particularly in machine translation. Unlike traditional lexical-based metrics such as BLEU or ROUGE, COMET leverages pre-trained multilingual language models to evaluate the semantic adequacy, fluency, and overall coherence of generated text. It operates by comparing the generated output to human references while also considering the source text. This feature makes it more robust in capturing meaning preservation and contextual accuracy. In the context of clinical note summarization, COMET provides a more nuanced evaluation by assessing how well the generated summaries align with reference summaries in terms of both linguistic and semantic quality [41].

#### BERTScore

BERTScore is a semantic evaluation metric that measures similarity between generated and reference texts using pretrained BERT embeddings. Unlike BLEU or ROUGE, which rely on exact matches, BERTScore computes cosine similarity between word embeddings and captures meaning even when words are rephrased or replaced by synonyms [42]. The BERTScore formula is calculated as follows:

$$\text {BERTScore} = \frac{1}{N} \sum _{i=1}^{N} \max _{j} \text {cosine similarity}(\textbf{h}_i, \textbf{h}_j)$$

where $\textbf{h}_i$ represents the *embedding vector* of a word in the generated summary, and $\textbf{h}_j$ is the *embedding vector* of a word in the reference summary. The score is computed by taking the highest similarity for each word in the generated summary, ensuring that *semantic equivalents* are recognized even if the exact words do not match.

## Results

In this section, we present the results of our experiments, where we utilized 16 LLMs from various companies, including OpenAI, DeepSeek, Meta, Google, Mixtral, and Alibaba, to generate journal summaries for 30,000 patients from the MIMIC-IV-Note. Each clinical note containing an average of 1,602 words. To evaluate the quality of the generated summaries, we employed six performance metrics. These metrics were carefully selected to cover various aspects of summary evaluation, such as n-gram overlap, word matching, semantic accuracy, and contextual correctness. By incorporating both precision-based measures (e.g., BLEU) and semantic-based measures (e.g., COMET and BERTScore), we achieved a comprehensive assessment of both extractive and abstractive summary generation. The results of our experiments for both approaches are presented in Tables 1 and 2. It is important to note that the reported values represent the “$\text {mean} \pm \text {standard deviation}$” calculated across 30,000 clinical notes.

### Extractive Approach

As seen in Table 1, Gemma-3-27B demonstrates strong performance across several key metrics, including BLEU (37.258) and BERTScore F1 (0.832), while leading in ROUGE-1 (0.736) and ROUGE-L (0.592), and achieving one of the highest METEOR scores (0.597). These results indicate Gemma-3-27B’s ability to generate high-quality summaries that capture both surface-level details and deeper contextual meaning. In terms of ROUGE-2, both Gemma-3-12B and DeepSeek-R1-70B reach the highest score ($\approx$ 0.53) that highlights their capacity to capture bi-gram-level lexical overlap which is critical for preserving intermediate textual structure. These values suggest solid performance, particularly in tasks where both lexical overlap and semantic understanding are crucial.

GPT-4-Turbo achieves the best performance in COMET (0.755) that indicates its semantic accuracy in clinical note summarization and its ability to preserve the intended meaning of the original text. GPT-o3-mini leads in BLEU (39.721) that demonstrates its strong capacity to match n-grams between the generated and reference summaries. This confirms GPT-o3-mini’s strength in lexical overlap, especially for tasks that requires precise word choices and surface-level accuracy.

On the other hand, DeepSeek models also show notable strengths. DeepSeek-R1-70B achieves the best ROUGE-2 score (0.529), emphasizing its capacity for capturing phrase-level overlaps. DeepSeek-R1-671B reaches the highest METEOR score (0.598), which reflects its alignment in both word order and semantic meaning. Furthermore, it also achieves the highest BERTScore Precision (0.897), along with Qwen-2.5-32B (0.898). This level of precision suggests strong semantic fidelity in the generated summaries. Although DeepSeek models do not lead across all metrics, their consistent strength and lower computational requirements make them suitable for resource-constrained applications.

LLaMA models, such as LLaMa-3-8B perform reasonably well, which shows decent efficiency with moderate summarization quality. They do not outperform Gemma, GPT, or DeepSeek models in any metric but remain practical for deployments with tighter resource budgets.

Mixtral-8x7B achieves high BERTScore Precision (0.870), indicating semantic accuracy, though it does not lead in any particular metric. It underperforms in ROUGE and BLEU, which suggests a trade-off in favor of deeper meaning over surface similarity. Qwen-2.5-32B, by contrast, achieves the best BERTScore Precision (0.898), which highlights its ability to maintain key semantic content. Qwen-3-32B leads in BERTScore Recall (0.814) and shows a high F1 (0.837), reflecting its capacity to capture the full meaning of reference summaries while maintaining strong overall fidelity. These models may be best suited for use cases where semantic completeness is prioritized over strict n-gram alignment, though their balance across other metrics is more variable.

With these results, it is clear that Gemma-3-27B demonstrates the most comprehensive performance across lexical and semantic metrics that presents it a top-performing model for extractive summarization. GPT models, particularly GPT-4o and GPT-4-Turbo, remain highly competitive due to their strong performance in COMET and BERTScore F1-score which are critical in clinical settings that requires accurate and fluent summaries. Gemma-2-9B maintains well-rounded performance across all evaluated metrics, offering a viable alternative for summarization tasks where a trade-off between quality and efficiency must be made.

### Abstractive Approach

The results in Table 2 highlight the strengths of various models in generating abstract summaries. It shows a more diverse distribution of top scores compared to the extractive approach.

LLaMa-3-8B achieves the highest ROUGE-1 score (0.435) that demonstrates its strength in capturing overall lexical overlap with the reference summaries. However, it does not perform as well in semantic-focused metrics such as COMET and BERTScore. It suggests more focus on surface-level matching. This makes LLaMa-3-8B suitable for applications where token-level alignment is more important than deep semantic representation.

Mixtral-8$\times$22B leads in ROUGE-2 (0.351). It indicates a strong ability to capture bi-gram level phrase structure. This shows the model’s effectiveness in preserving short-range dependencies and local coherence in generated summaries. However, its performance in semantic metrics like COMET and BERTScore is less competitive. It suggests a trade-off favoring structural alignment over deeper understanding.

GPT-4-Turbo performs best in ROUGE-L (0.368) and METEOR (0.313), which highlights its strong alignment with the longest common subsequences and fluency at the sentence level. These scores suggest that GPT-4-Turbo generates grammatically fluent and lexically coherent text, suitable for tasks where readability and format are key. However, it does not lead in semantic alignment metrics such as COMET or BERTScore, placing it slightly behind other models in terms of deeper understanding.

DeepSeek-R1-70B achieves the highest BLEU score (9.400), which indicates its superior capability in exact n-gram overlap, especially for short phrases. It also ties for the highest BERTScore Recall (0.810) and F1 (0.837), which shows it captures a broad range of semantic content while preserving fluency. This makes it one of the strongest models overall for abstractive summarization in contexts requiring both surface and semantic fidelity.

Qwen-3-32B, with the highest COMET score (0.718), shows the strongest agreement with human reference summaries in terms of semantic adequacy and fluency. It also achieves a good BERTScore F1 score (0.835), which was close to the best F1 score across the models. It shows its capacity to produce summaries that balance semantic correctness with expressive clarity. These results make Qwen-3-32B highly suitable for tasks focused on faithful representation of meaning, even without strict lexical overlap.

Gemma-3-27B performs exceptionally well across semantic metrics with Precision (0.867), Recall (0.806), and F1 score (0.835), which is close performance to the highest BERTScore. This model shows a strong ability to retain core information and semantic fidelity in the generated summaries. Although it does not lead in BLEU or ROUGE, its consistency across semantic benchmarks indicates its strength in understanding and reproducing complex textual content.

Gemma-2-9B also has a BERTScore Precision close to the best (0.865), which shows accurate semantic focus and strong alignment in meaning. However, its performance in lexical metrics such as BLEU and METEOR is lower, which suggests a prioritization of meaning over surface form. This model may be suitable where interpretation matters more than wording.

GPT-4o ties for the highest BERTScore Precision (0.873), a result that shows its summaries are semantically precise. While its ROUGE and BLEU scores are lower, this metric supports its use in clinical or critical tasks where the exact interpretation of information is essential. GPT-4o remains a strong option when semantic clarity and contextual coherence are prioritized.

Other models such as Mixtral-8x7B and Qwen-2.5-32B demonstrate reasonable performance in BERTScore, but do not lead in any individual metric. Their results suggest a focus on semantic accuracy, though with less balance across other dimensions. These models may serve niche applications where specific trade-offs are acceptable.

Overall, DeepSeek-R1-70B, Gemma-3-27B, and Qwen-3-32B emerge as the top-performing models across both lexical and semantic metrics, depending on whether fluency, precision, or meaning preservation is prioritized.

**Table 1:** Extractive summarization performance across models. Bold values indicate the highest performance for each metric. Bold values indicate the highest performance for each metric

Model (#Parameters)	ROUGE-1	ROUGE-2	ROUGE-L	BLEU	COMET	METEOR	BERTScore		
							Precision	Recall	F1
GPT-o1-mini	0.651±0.064	0.425±0.111	0.454±0.107	36.304±12.568	0.717±0.046	0.556±0.113	0.859±0.013	0.776±0.003	0.821±0.010
GPT-o3-mini	0.642±0.158	0.478±0.200	0.507±0.194	**39.721**±19.766	0.720±0.043	0.587±0.217	0.868±0.018	0.762±0.008	0.811±0.012
GPT-4-Turbo	0.672±0.110	0.506±0.160	0.514±0.161	24.823±4.731	**0.755**±0.036	0.589±0.167	0.870±0.012	0.788±0.006	0.823±0.006
GPT-4o	0.731±0.012	0.519±0.137	0.579±0.122	30.651±1.234	0.742±0.033	0.589±0.114	0.882±0.002	0.797±0.004	**0.841**±0.009
DeepSeek-R1-671B	0.561±0.091	0.307±0.110	0.323±0.112	19.987±13.544	0.724±0.029	**0.598**±0.145	0.897±0.007	0.784±0.004	0.831±0.006
DeepSeek-R1-70B	0.687±0.140	**0.529**±0.190	0.549±0.180	33.690±18.950	0.744±0.030	0.513±0.210	0.864±0.010	0.793±0.009	0.827±0.009
LLaMa-3.3-70B	0.575±0.056	0.354±0.086	0.380±0.065	20.070±8.225	0.733±0.037	0.378±0.084	0.843±0.005	0.788±0.007	0.831±0.006
LLaMa-3-8B	0.535±0.104	0.522±0.175	0.517±0.152	31.142±18.373	0.723±0.039	0.554±0.152	0.835±0.009	0.776±0.003	0.828±0.007
Gemma-3-27B	**0.736**±0.173	0.519±0.135	**0.592**±0.189	37.258±12.469	0.746±0.022	0.597±0.242	0.871±0.012	0.796±0.003	0.832±0.006
Gemma-3-12B	0.675±0.117	0.528±0.162	0.567±0.113	35.599±9.772	0.744±0.022	0.581±0.174	0.866±0.017	0.771±0.004	0.816±0.008
Gemma-2-9B	0.686±0.126	0.515±0.186	0.560±0.208	36.058±16.883	0.743±0.035	0.573±0.216	0.842±0.017	0.769±0.006	0.823±0.001
Mixtral-8$\times$22B	0.622±0.172	0.419±0.211	0.409±0.189	25.776±14.281	0.725±0.037	0.449±0.119	0.877±0.016	0.788±0.008	0.830±0.008
Mixtral-8$\times$7B	0.574±0.134	0.376±0.170	0.411±0.165	20.792±17.922	0.727±0.041	0.395±0.187	0.870±0.010	0.768±0.007	0.816±0.010
Qwen-2.5-32B	0.628±0.140	0.469±0.160	0.475±0.160	27.390±18.680	0.750±0.030	0.428±0.180	**0.898**±0.011	0.793±0.010	0.828±0.010
Qwen-3-32B	0.665±0.160	0.483±0.171	0.488±0.137	26.721±15.834	0.753±0.027	0.449±0.139	0.871±0.006	**0.814**±0.002	0.837±0.003
Qwen-3-8B	0.663±0.111	0.432±0.176	0.471±0.208	26.666±11.784	0.729±0.021	0.443±0.004	0.874±0.004	0.771±0.007	0.820±0.004

**Table 2:** Abstractive summarization performance across models. Bold values indicate the highest performance for each metric. Bold values indicate the highest performance for each metric.

Model (#Parameters)	ROUGE-1	ROUGE-2	ROUGE-L	BLEU	COMET	METEOR	BERTScore		
							Precision	Recall	F1
GPT-o1-mini	0.324±0.056	0.136±0.029	0.194±0.046	1.673±2.064	0.677±0.036	0.180±0.067	0.850±0.012	0.757±0.003	0.801±0.007
GPT-o3-mini	0.370±0.053	0.205±0.062	0.269±0.047	2.199±2.190	0.670±0.031	0.213±0.073	0.861±0.001	0.759±0.005	0.807±0.003
GPT-4-Turbo	0.418±0.066	0.343±0.059	**0.368**±0.069	3.314±1.791	0.695±0.036	**0.313**±0.050	0.860±0.013	0.757±0.001	0.805±0.011
GPT-4o	0.364±0.087	0.191±0.071	0.236±0.073	3.202±3.543	0.712±0.024	0.196±0.079	**0.873**±0.012	0.803±0.007	0.810±0.008
DeepSeek-R1-671B	0.399±0.063	0.298±0.071	0.366±0.035	5.694±1.760	0.710±0.035	0.200±0.026	0.855±0.003	0.782±0.005	0.817±0.002
DeepSeek-R1-70B	0.386±0.100	0.257±0.080	0.286±0.090	**9.400**±4.810	0.714±0.030	0.213±0.070	0.864±0.010	**0.810**±0.010	**0.837**±0.001
LLaMa-3.3-70B	0.355±0.092	0.251±0.098	0.286±0.101	2.968±3.015	0.678±0.030	0.208±0.070	0.860±0.010	0.785±0.010	0.821±0.010
LLaMa-3-8B	**0.435**±0.088	0.285±0.089	0.298±0.096	4.776±3.168	0.683±0.022	0.232±0.074	0.847±0.035	0.792±0.020	0.814±0.001
Gemma-3-27B	0.402±0.071	0.244±0.085	0.281±0.073	4.538±.846	0.697±0.025	0.237±0.041	0.867±0.009	0.806±0.019	0.835±0.008
Gemma-3-12B	0.414±0.071	0.271±0.073	0.295±0.079	5.145±3.334	0.704±0.023	0.226±0.066	0.857±0.014	0.804±0.011	0.830±0.007
Gemma-2-9B	0.425±0.084	0.319±0.097	0.345±0.091	1.960±5.820	0.688±0.041	0.221±0.079	0.865±0.009	0.781±0.012	0.821±0.008
Mixtral-8$\times$22B	0.413±0.105	**0.351**±0.008	0.348±0.210	4.731±6.147	0.688±0.045	0.249±0.099	0.863±0.015	0.797±0.004	0.828±0.007
Mixtral-8$\times$7B	0.428±0.109	0.335±0.118	0.346±0.123	2.780±8.223	0.682±0.043	0.218±0.108	0.864±0.019	0.768±0.008	0.813±0.011
Qwen-2.5-32B	0.353±0.090	0.216±0.070	0.247±0.080	3.577±3.240	0.698±0.030	0.186±0.060	0.864±0.010	0.793±0.010	0.827±0.010
Qwen-3-32B	0.373±0.011	0.291±0.043	0.243±0.057	4.483±2.763	**0.718**±0.044	0.211±0.055	0.857±0.008	0.804±0.013	0.835±0.008
Qwen-3-8B	0.311±0.019	0.222±0.031	0.227±0.042	3.217±2.755	0.677±0.036	0.214±0.073	0.855±0.009	0.801±0.017	0.827±0.007

### Cost and Time Complexity

Another crucial consideration in deploying LLMs in practical settings, such as the clinical domain, is the associated cost and response time. Real-time processing is essential for many applications, particularly in clinical environments. To address this, we conducted an experiment to evaluate the average response time and cost for each LLM when processing a single patient’s journal entry. Additionally, considering the data protection regulations and privacy concerns, it is important in many countries that patient information remains confidential and is not publicly accessible. Therefore, we also included information about the potential for using these LLMs locally to ensure data security, as shown in Table 3.

**Table 3:** Model performance comparison

Model (#Parameters)	Time/Note (s)		Cost/Note ($)	Server
	Cloud$$^{a}$$	A100$$^{b}$$		
GPT-o1-mini	52.36	–	0.007	Only Cloud
GPT-o3-mini	46.68	–	0.007	Only Cloud
GPT-4-Turbo	18.56	–	0.055	Only Cloud
GPT-4o	10.98	–	0.015	Only Cloud
DeepSeek-R1-671B	68.23	–	0.002	Local/Cloud
DeepSeek-R1-70B	25.31	715.6	Free	Local/Cloud
LLaMa-3.3-70B	22.76	691.1	Free	Local/Cloud
LLaMa-3-8B	–	5.49	Free	Local/Cloud
Gemma-3-27B	–	22.21	Free	Local/Cloud
Gemma-3-12B	–	13.56	Free	Local/Cloud
Gemma-2-9B	–	8.04	Free	Local/Cloud
Mixtral-8$$\times$$22B	–	389.2	Free	Local/Cloud
Mixtral-8$$\times$$7B	–	7.83	Free	Local/Cloud
Qwen-2.5-32B	–	25.39	Free	Local/Cloud
Qwen-3-32B	–	37.49	Free	Local/Cloud
Qwen-3-8B	–	15.86	Free	Local/Cloud

$^{a}$ Time measured for the models on a cloud platform $^{b}$ Time measured for the models on a local server with one A100 GPU (40 GB)

As shown in Table 3, the fastest models for summarization are LLaMa-3-8B, Mixtral-8x7B, and Gemma-2-9B, which return results in less than 10 seconds. Moreover, Gemma-3-12B and Qwen-3-8B models prepare the results in a time range of 10 to 20 seconds. These models are ideal for real-time applications where fast response times are essential. Additionally, they can be deployed locally and are free of charge, which makes them suitable for offline applications that require strict security policies and data privacy.

Following these models, the GPT-4 series can generate summaries within 10 to 20 seconds, but they are restricted to cloud usage and come with an associated cost of $0.015 and $0.055 per note with approximately 1,602 words. The next group of models includes DeepSeek-R1-70B, LLaMa-3.3-70B, Gemma-3-27B, Qwen-3-32B, and Qwen-2.5-32B, which are also free and can be deployed locally. However, it is important to note that DeepSeek-R1-70B and LLaMa-3.3-70B are highly resource-intensive. When executed locally on an A100 GPU, their response times rise sharply from about 25.31 and 22.76 seconds to 715.6 and 691.1 seconds, respectively.

Following these, GPT-o1-mini and GPT-o3-mini exhibit response times of approximately 50 seconds per note and cost $0.007 per note, but they can only be used in cloud environments. Lastly, DeepSeek-R1-671B has a response time of 68.39 seconds per note, and although it can be deployed either locally or on the cloud, it is the most affordable option among the cloud-based models.

## Discussion

In the medical domain, there is a growing body of research and real-world implementations where hospitals and clinical institutions leverage LLMs for specific tasks in clinical practice [13–18]. The ongoing advances in AI suggest that we will continue to witness even more impactful applications of these tools in the healthcare sector in the near future. Through continuous dialogue with clinicians about the deployment of AI in medical practice, one of the most frequently highlighted and promising applications is the summary of clinical notes.

Taking into account the importance of efficient clinical note summaries, our study evaluated leading LLMs from major AI companies using the MIMIC-IV-Note data. By assessing the performance of these models, we aim to provide insights into their effectiveness in processing medical text, ultimately contributing to advances in AI-driven healthcare solutions.

### Applications and Model Selection

The choice of an appropriate LLM for clinical summarization depends on balancing quality, speed, cost, and deployment feasibility. In the extractive setting, Gemma-3-27B stands out with the best overall lexical and semantic performance, while DeepSeek-R1-70B and GPT-4-Turbo also show strong results in ROUGE-2 and COMET, respectively. GPT-o3-mini excels in BLEU, and Qwen and DeepSeek models achieve the highest BERTScore Precision, highlights their semantic fidelity.

For abstractive summarization, performance is more distributed. LLaMa-3-8B leads in ROUGE-1, Mixtral-8x22B in ROUGE-2, GPT-4-Turbo in ROUGE-L and METEOR, and DeepSeek-R1-70B in BLEU and BERTScore F1 score. Qwen-3-32B delivers the highest COMET score, while Gemma-3-27B and GPT-4o provide consistently strong semantic metrics. These results confirm that abstractive summarization remains more challenging, but several models demonstrate competitive performance depending on the priority of lexical overlap, fluency, or semantic adequacy.

Efficiency and deployment constraints also shape model selection. Smaller models such as LLaMa-3-8B, Mixtral-8x7B, and Gemma-2-9B return results in under 10 seconds locally and at no cost, making them suitable for real-time or privacy-sensitive use. In contrast, larger models like DeepSeek-R1-70B and LLaMa-3.3-70B demand extensive hardware resources, with runtimes exceeding 700 seconds on local GPUs. GPT-4o achieves strong semantic quality and the fastest inference among cloud models but incurs usage costs and lacks local deployability.

Overall, Gemma-3-27B offers the best trade-off between quality and local deployment, while GPT-4o remains a strong option when cloud use is acceptable. Importantly, our findings show that larger parameter sizes do not always guarantee better performance, as lightweight models such as LLaMa-3-8B and Gemma-2-9B often deliver comparable results with lower cost and faster runtimes.

### Clinical Considerations and Prospective

In many countries, patient health data is classified as sensitive or confidential, which prevents it from being made publicly available. Beyond technical performance, clinical deployment of LLMs must therefore comply with strict data protection regulations such as the European Union’s General Data Protection Regulation (GDPR), the United States’ Health Insurance Portability and Accountability Act (HIPAA), and China’s Personal Information Protection Law (PIPL). These frameworks often mandate that sensitive data remain within secure environments, which creates challenges for cloud-only models such as GPT variants. While cloud APIs offer strong performance and easy access, they also raise compliance concerns. They require reliance on third-party infrastructure and create recurring usage costs.

In contrast, locally deployable models give greater control over data and infrastructure and provide a practical solution for privacy-sensitive healthcare settings. Open-source models such as Gemma, LLaMA, Mixtral, and Qwen can be run locally at no licensing cost. This makes them attractive for institutions with strict security requirements. Cloud-based models like GPT-4o remain valuable when semantic accuracy is critical and external processing is permissible, but they may be limited by regulatory restrictions.

Overall, clinical adoption of LLMs must weigh not only accuracy and efficiency but also compliance, data governance, and deployment flexibility. Future progress will depend on aligning model capabilities with healthcare-specific privacy frameworks to ensure safe and practical integration into clinical workflows.

### Limitations and Future Studies

This study offers several strengths, including a broad comparison of LLMs across performance, efficiency, and cost. By evaluating both extractive and abstractive approaches, it provides a solid view of model capabilities in clinical summarization. Clinician input for prompt refinement further ensures practical relevance.

Despite these strengths, there are limitations. First, the evaluation relied on automatic metrics such as ROUGE and BLEU, which emphasize surface-level lexical overlap and cannot fully capture semantic coherence. While advanced metrics like COMET and BERTScore address some of these shortcomings by focusing on semantic equivalence, they too fall short of reflecting the nuanced judgment of clinicians, especially regarding medical terminology and context-specific meaning.

Second, there is currently no large-scale clinician-annotated dataset for clinical note summarization. Automatic metrics are therefore the only practical option for evaluating performance at scale. These metrics are useful but cannot replace expert human judgment. In this study, clinicians refined prompts iteratively but did not systematically assess the generated summaries. A structured framework for clinician-led evaluation is essential to ensure the medical accuracy, relevance, and practical utility of LLM-generated summaries.

Future studies should address the lack of large clinician-annotated datasets for clinical note summarization. Such resources would allow more reliable training and evaluation beyond automatic metrics. There is also a need for domain-specific metrics that capture semantic accuracy and medical context while remaining interpretable for clinicians. Research should develop systematic methods to detect and reduce hallucinations and combine automated tools with clinician oversight to ensure factual reliability and patient safety. Future work should also integrate multidimensional evaluation criteria such as fluency, coherence, and consistency together with structured clinician feedback to provide a more holistic assessment of model performance.

## Conclusion

This study benchmarked 16 LLMs for extractive and abstractive summarization of MIMIC-IV-Note clinical notes. The performance is evaluated across lexical and semantic metrics as well as runtime, cost, and deployment feasibility. The results show that no single model dominates across all dimensions. Gemma-3-27B demonstrated the strongest overall performance for extractive summarization. On the other hand, DeepSeek-R1-70B, Qwen-3-32B, and GPT-4o were leading candidates for abstractive summarization depending on whether lexical overlap, semantic adequacy, or fluency was prioritized. Moreover, larger parameter sizes did not always yield better performance, as lightweight models such as LLaMa-3-8B and Gemma-2-9B often delivered comparable results with much faster runtimes and lower computational costs.

From a deployment perspective, local open-source models such as Gemma, LLaMA, Mixtral, and Qwen offer flexibility and privacy at no licensing cost. This makes them attractive for institutions with strict data governance requirements. Cloud-only models such as GPT-4o provide strong semantic accuracy and fast inference but raise concerns about regulatory compliance and recurring usage costs.

Overall, this study highlights the trade-offs between model quality, efficiency, and deployment context. Clinical adoption of LLMs for summarization will depend on aligning technical performance with practical requirements such as privacy, cost-effectiveness, and integration into existing workflows. Future research should focus on building large clinician-annotated datasets, developing domain-sensitive evaluation metrics, and designing frameworks that combine automated scoring with structured human feedback to ensure that LLM-generated summaries are accurate, reliable, and clinically meaningful.

## Data Availability

The original data are available in the MIMIC repository, https://physionet.org/content/mimic-iv-note/2.2/
